# Central Serous Chorioretinopathy Mistaken for Tuberculous Choroiditis

**Published:** 2011-10

**Authors:** Marina Papadia, Carl P. Herbort

**Affiliations:** 1 Inflammatory and Retinal Eye Diseases, Center for Ophthalmic Specialized Care, Lausanne, Switzerland; 2 Eye Clinic, Department of Neurosciences, Ophthalmology and Genetics, University of Genova, Genova, Italy; 3 University of Lausanne, Lausanne, Switzerland

**Keywords:** Central Serous Chorioretinopathy, Tuberculosis, Serous Detachment, Corticosteroid Therapy

## Abstract

**Purpose:**

To report a patient erroneously diagnosed with tuberculous choroiditis who was accordingly treated with long term steroids which in turn, worsened the actual disease process that turned out to be central serous chorioretinopathy (CSC).

**Case Report:**

A 59-year-old Caucasian man developed a chorioretinal disease in his right eye in 1997. Having a positive tuberculin skin test, tuberculous chorioretinitis was suspected and antituberculous therapy was administered for 4 months. In 2005, visual symptoms in the same eye recurred and despite negative interferon gamma release assay, tuberculous choroiditis was considered as the diagnosis and the patient further received massive corticosteroid therapy along with antituberculous agents. Despite a deteriorating clinical picture, therapy was continued. Upon initial examination at our center, no sign of inflammation was observed and a diagnosis of CSC was made, consequently steroid therapy was terminated.

**Conclusion:**

In some chorioretinopathies, it is difficult to differentiate inflammatory from non-inflammatory causes. One should observe the course of the disease and question the initial diagnosis when no improvement or deterioration occurs despite therapy.

## INTRODUCTION

Central serous chorioretinopathy (CSC) is a condition which may be exacerbated by corticosteroids in susceptible individuals.^1^ When affected patients are referred to uveitis clinics, the condition runs the risk of being assimilated as an inflammatory condition. CSC is not completely understood; it has systemic associations, a multifactorial etiology and a complex pathogenesis. Increased permeability of choroidal vessels is thought to cause focal or diffuse leakage under the retinal pigment epithelium (RPE) resulting in neurosensory retinal dysfunction. Detachments of the neurosensory retina are usually associated with multiple small or large RPE detachments which are quasi diagnostic when present.^2^

In the case of certain chorioretinopathies, distinguishing inflammatory from non-inflammatory causes is rather difficult. One of the entities that can mimic an inflammatory chorioretinopathy is CSC or its more aggressive/chronic form, diffuse retinal pigment epitheliopathy.

It is critical to identify CSC or its chronic form in patients referred to uveitis centers otherwise, there is a real danger that the patient be treated with corticosteroids which are likely to aggravate the disease and cause irreversible damage.

Development of CSC following administration of corticosteroids by a variety of routes is a well-known phenomenon.^3^ Herein, we report acute visual loss after receiving systemic corticosteroids in a patient whose CSC was initially misinterpreted as tuberculous choroiditis and was treated with antituberculous chemotherapy combined with systemic corticosteroids despite repeated negative interferon gamma releasing assays (IGRAs), leading to progressive worsening of the condition over a ten-year period.

## CASE REPORT

A 59-year-old Caucasian man was diagnosed with a non-specified chorioretinal disorder in his right eye back in 1997; at that time he received antituberculous chemotherapy for 4 months due to a positive tuberculin skin test (12 by 16 mm induration). A recrudescence of psoriasis, which the patient used to treat with corticosteroid ointments, had preceded the ocular symptoms. In 2005, after another exacerbation of psoriasis, visual symptoms recurred in the same eye. Uveitis work-up showed only a positive tuberculin skin test considering the fact that he had received BCG (Bacillus Calmette-Guerin) vaccination. Tuberculous chorioretinitis was suspected once more and antituberculous quadri-therapy together with systemic corticosteroids was initiated despite a negative IGRA test (QuantiFERON-TB Gold In-Tube, Cellestis, Carnegie, Australia). This test identifies latent or active tuberculous infection by detecting memory T-cells reacting against *Mycobacterium tuberculosis* proteins. It measures the release of interferon gamma in response to antigens of this certain type of bacterium but not to the BCG vaccine nor to most non-tuberculous mycobacteria. Despite this therapy, visual acuity worsened.

In 2010, when the patient attended our center for a second opinion, he was still on low-dose systemic corticosteroids. Best corrected visual acuity (BCVA) was 0.05 and 1.0 in the right and left eyes respectively. Both anterior segments were normal and laser flare photometry (Kowa FM-500, Kowa Ltd., Tokyo, Japan) measured slight subclinical disruption of the blood-aqueous barrier in both eyes (9.7 and 9.4 ph/ms in the right and left eyes respectively). Intraocular pressure was 14 mmHg in both eyes. On fundus examination, rare pigmented cells were found in the vitreous body of the right eye, along with widespread chorioretinal scars in both eyes.

**Figure 1:**
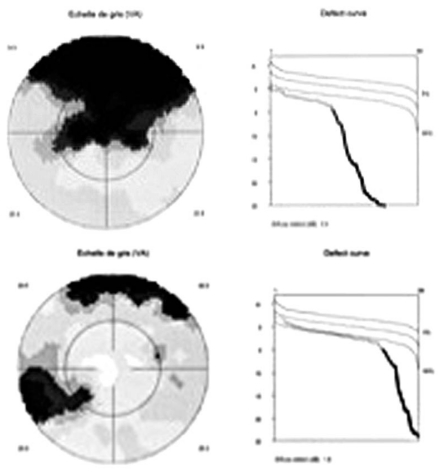
Visual field testing shows severe loss of the superior visual field in the right eye (upper image) and an absolute scotoma in the left one (lower image).

Visual field testing (G1 program of the Octopus 900, G Standard; Haag-Streit International, Bern, Switzerland) demonstrated severe loss of the superior visual field in the right eye and an absolute superior scotoma in the left eye; both field defects corresponded to chorioretinal lesions visible on fundus examination (Fig. 1).

**Figure 2:**
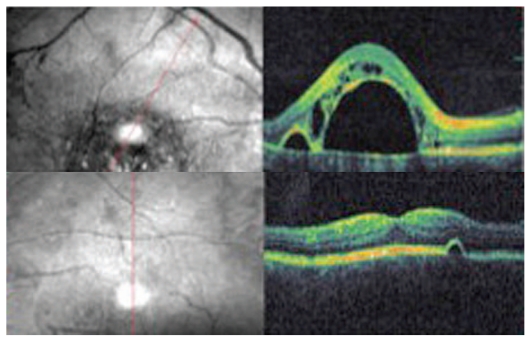
OCT images show a large RPE detachment adjacent to a smaller one together with intraretinal edema in the right eye (top image), and a very small serous RPE detachment in the left eye (bottom image).

OCT (OTI Spectral OCT/SLO; Ophthalmic Technologies Inc., Toronto, Canada) scan showed a large serous RPE detachment adjacent to a smaller one in the right eye, and a minute serous RPE detachment in the left eye (Fig. 2).

**Figure 3:**
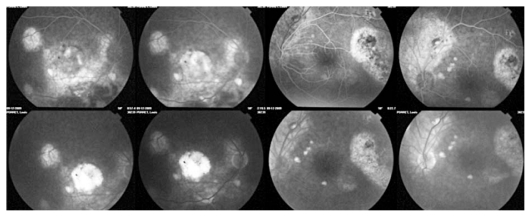
Fluorescein angiography demonstrates hyperfluorescent areas corresponding to exudation and/or chorioretinal scars without any sign of vasculitis or inflammation.

On fluorescein angiography (FA) hyperfluorescent areas were seen, indicating exudation and atrophy corresponding to the chorioretinal lesions observed on fundus examination, while no signs of inflammation or vasculitis were present (Fig. 3).

**Figure 4:**
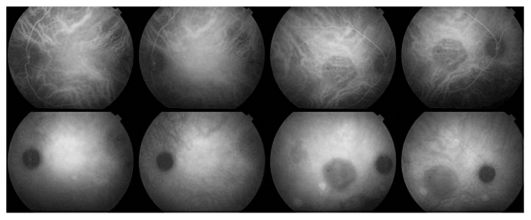
ICGA shows a round dark area corresponding to the right RPE detachment and late diffuse posterior pole hyperfluorescence.

Indocyanine green angiography (ICGA) revealed a round dark area, corresponding to the large exudative right RPE detachment and also late diffuse choroidal hyperfluorescence in both eyes as can usually be found in patients with CSC. The dark area was caused by capillary closure due to pressure exerted by the RPE detachment, or by masking effect because of increased liquid density in the RPE detachment (Fig. 4).

**Figure 5:**
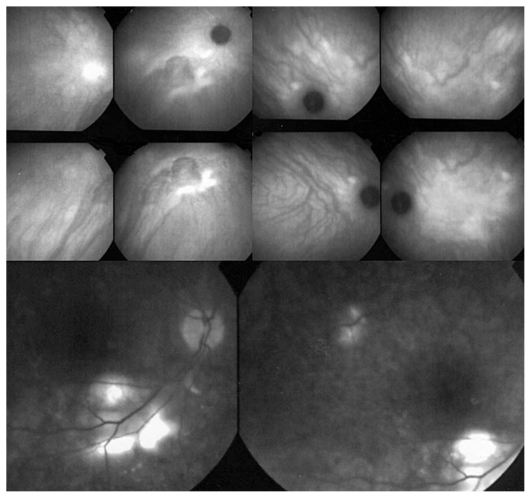
FA and ICGA frames reclaimed from the former treatment center. FA (bottom frames) shows no signs of an inflammatory condition whereas, ICGA (top eight frames) shows signs clearly compatible with CSC, especially bilateral late diffuse choroidal hyperfluorescence.

Based on these findings, a diagnosis of CSC was made and corticosteroid therapy was discontinued. Earlier ICGA and FA images (when the disease had first appeared in 1997) were requested from the center that had taken care of the patient. FA showed no signs of inflammation, while ICGA frames showed areas of diffuse late choroidal hyperfluorescence, a finding typical for CSC (Fig. 5).

After 6 months, OCT, FA and ICGA remained unchanged as did visual acuity. Visual field improvement was the only sign, if any, of recovery.

## DISCUSSION

The deleterious effects of corticosteroids in terms of predisposing to or aggravating CSC are widely recognized.^4^ This issue should be borne in mind when unexpected clinical and angiographic evolution compatible with CSC occurs in a putative uveitis patient treated with corticosteroids.

The long-term visual prognosis of CSC is fair without treatment, but a significant proportion of patients develop recurrences.^5^ FA and ICGA, along with OCT scans are essential tools in the diagnosis of both inflammatory eye diseases and CSC.^6^

Foveal attenuation, chronic macular edema, and damage to the photoreceptor layer have been reported as causes of visual loss in CSC. Photoreceptor atrophy in the fovea, despite successful retinal reattachment, typically occurs when the duration of symptoms exceeds 4 months.^7^

It should be mentioned that although the incidence of tuberculous choroiditis has increased in the past decades, its diagnosis should be supported by a positive IGRA test.^8^ This seems to have been one of the major pitfalls in the management of this patient.

The possibility of CSC development in uveitis patients should be kept in mind as an adverse effect of corticosteroid therapy and should not be mistaken as worsening of uveitis resulting in an erroneous increase in intensity of steroid therapy.^3^

The case described herein demonstrates how an incorrect inflammatory diagnosis made at the beginning and later, was never doubted during follow-up visits leading to irreversible retinal damage caused by aggravation of CSC due to corticosteroids.
